# From Text on Paper to Digital Poetry: Creativity and Digital Literary Reading Practices in Initial Teacher Education

**DOI:** 10.3389/fpsyg.2022.882898

**Published:** 2022-06-17

**Authors:** Moisés Selfa Sastre, Enric Falguera Garcia

**Affiliations:** Department of Specific Didactics, Universitat de Lleida, Lleida, Spain

**Keywords:** digital literature, digital practices, cyberpoetry, creativity, digital writing, teachers

## Abstract

The new contexts of literary education allow for the creation of digital reading and writing practices related to what specialised literature calls digital literature. Among these practices and with an eminently theoretical content and with an example of this content, in this paper, we want to focus our gaze on cyberpoetry, conceived as an exercise in literary creativity that firstly involves use of technology and specific software for the digital creation of poetic texts and, last but not least, knowledge and mastery of poetic language and the literary conventions linked thereto. From this point of view, in initial teacher training, we work with future teachers to create cyberpoems with a dual purpose: on the one hand, to reflect on what literary reading in digital format entails and to rehearse reading mediation processes that can be carried out with this type of literature, and, on the other, to begin in the digital creation of cyberpoems that, later, may be presented and worked in a real context of the school classroom. In this paper, we present digital practices of literary reading that have been created by student teachers in initial training. The creation of these practices has been carried out with the *Genially* tool. For data collection, the URL of each creation is accessed in order to analyse them. The analysis of the data follows the parameters of the qualitative methodology, specifically based on three categories of analysis for each digital creation: multimodality, hypertextuality and the interaction of each digital reading proposal. The conclusions of all this allow us to affirm that the creation of cyberpoems is an exercise in literary creativity that has to take into account the digital dimension of the literary text and its reading comprehension in a multimodal environment. Thus, the teacher in initial training carries out a digital literary mediation exercise, of a creative type, which he will later carry out in his pedagogical practice.

## Introduction

Literature, i.e., the art of written or spoken expression, is an artistic manifestation that every cultural community has cultivated throughout ages. As such, it is no stranger to advances in civilisations that have been imposed due to many factors. Two can be generalised among them: the development of human thought and the technological progress associated with it (Solbes and Traver, 2015). From this point of view, literature has posed a challenge to the reader, especially since the discovery of the printing press and, therefore, of the transmission of literary text from a printed medium, which can be summarised as follows: the author expects a response from his potential receiver, which is the object of study of literary criticism (Chiappe, 2015).

Today we are immersed in a markedly digital age, where technological advances have expanded the author’s relationship with the reader of his text (Handayani et al., 2020: 65–74). Therefore, the questions we ask ourselves are twofold: how the reader, from a markedly technological environment, understands literature and how he sees it, and whether there are substantial changes, inherited from written culture, in the way of relating to the literary text. In order to approach these issues, which have a conceptual and markedly epistemological basis, we will focus on the field of literary reading and, more specifically, on digital literature, understood as those literary works created specifically for the digital format—mainly, but not only, for the Internet—, and which could not exist outside of it (Bootz, 2021: 7–22). It is not digital literature what is thus found in a virtual library, nor, strictly speaking, the linear narrative that is created for the Internet, since that same literature could be presented in book format. The interaction of the reader with the literary text based on the possibilities enabled by technology is what defines digital literature.

To this end, we establish three questions that we want to develop in their most fundamental features, namely, approaching the context of digital culture in which reading and literature are clearly inserted; identifying the parameters that allow us to underline creativity as the basis and sustenance of the reading of digital texts; and, finally, defining the variables of the creation of digital texts that imply a very particular reading of these texts. All the foregoing aims at advancing in the strategies for understanding and training readers and writers in the context of digital literature.

## Literature in the Digital Culture Sphere

Literature, understood as the art of the word, generates discourses of all kinds. And not only does it generate them, but it is also connected to such discourses, as they are all interrelated to each other through relations of continuity, opposition and provocation that link and oppose them (Morales-Sánchez and Martin-Villarreal, 2019). Digital literature has also changed the traditional relationship between the author of a text and its reader to the point of almost eliminating the role of the publisher at a stroke. This traditional triangle formed by author, reader and publisher is modified in a literate context dominated by digital culture, due to the near disappearance of the publisher, a fact that allows for simultaneous, reciprocal and not at all deferred communication between the author and the reader of the literary text (Cordón García, 2016).

The problem is no longer whether it will be possible to read literary texts on paper in 100 years’ time, but whether it will make sense to read them on paper at all. Therefore, when thinking about digital literature, is would be logic to focus on the dialogical strategies involved in thinking digital (Engen, 2019: 9–18). When all the foregoing is approached from literary discourse, an author’s approach to writing a literary text or the process of reading it takes on a truly interesting magnitude. Therefore, both reading and generating literature from this new digital space are essential to understanding digital literature and the concept of reading and creation that contextualises it (Pajares, 2015).

Focusing on the aspects that define the habits of writing and reading digital literary texts, it is necessary to highlight how digital literature has undeniably evolved at the vertiginous pace at which technology is also changing.

In the light of this, digital literary discourse demands an active response from the reader and an interaction with the literary work in which the classical hierarchical relations between author and reader are broken. Consequently, when we ask ourselves how, the way of constructing and receiving the literary text—as an aesthetic discourse—has changed in terms of literary communication, we see significant changes not only in the ways of conveying information, but also in articulating the mode of persuasion, as the focus is no longer only on the pure content of the literary message (Alghadeer, 2014: 87–96). It is also in the form it takes. We cab then logically think that each historical-cultural stage has its own way of creating and persuading according to its own technological domain and the dimensions of the reading masses, who are the recipients of literary discourse.

Nowadays, digital culture, which includes literature as a digital form of expression, brings infinite communicative possibilities demanding new ways of reading and creating in line with the digital and technological divide (Kangasharju et al., 2021: 52–91). All this is reflected in the most current digital literature in the form of artistic proposals, which convey the hybridity, versatility and complexity of new ways of understanding and interpreting literary discourse.

## Digital Literature, Literary Reading and Creativity: An Essential Trinomial

Reading a given literary text involves the effort to construct the meaning of a message with one or more aesthetic intentions. Hence, literary reading is specifically understood as an intellectual process whose essential principle is that of literary construction. By literary construction, we do not only understand the act of writing for aesthetic purposes, but we must also understand how reading requires a similar, and sometimes greater, effort than creating.

Digital literature presents the reader with a set of texts in which the word is not only the ultimate goal of reading, but also broadens its horizons towards other analogue, visual and sound codes, thus allowing experimentation linked to the technique, the software and the digital possibilities surrounding the literary message (Torres and Côrtes, 2021: 3–26). All the foregoing means that the process of constructing the digital text escapes the variables usually handled by who is reading the text. One of the singularities of digital authorship is its markedly collective nature, as writers, designers, computer scientists, audiovisual technicians and among others often join hands in a choral exercise of harmony in which the common denominator is creativity (Saum-Pascual, 2018: 34). Hence, the confluence of different creation nodes that transcend literary creativity as it has been conceived until a few days ago. We can therefore speak of a polyphonic discourse where creativity symbiosis allows the reader to grasp different perspectives and simultaneous aesthetic approaches.

From this point of view, it is coherent, following the approaches of Bakhtin’s concept of aesthetic polyphony, to speak of polyacroasis as the basis and foundation of digital literary texts, given that readers belong to different cultural and digital spaces and, therefore, with divergent reading habits and intertexts (Navarro-Romero, 2021: 75–81). The range of readings that the same digital text can suggest allows us to speak of the concept of reading-writing (Llosa, 2019: 105–112), i.e., the possibility of handling the text not only in the reader’s intellect, but the reader takes on the role of creator by being able to intervene in the literary message directly. We are thus talking about someone who produces his own texts almost simultaneously with their reception.

This approach leads us to affirm that the authorship of the digital text can also lie with the reader. Thus, three more factors in this writing process should be considered: firstly, the vision of the reader as a player who develops strategies and techniques derived from the multimedia world; secondly, the identification of the reader as an actor who simultaneously constructs and deconstructs a pattern of reading and writing that must be seen while selecting the tools to do so and accessing the meaning of the digital text; and thirdly, digital literature has a markedly artistic vein in which the writer resorts in parallel to a double code, that of literary language and that of computer software, in such a way that the presence of technology in the parameters of creation is indisputable: That of literary language and that of computer software, in such a way that the presence of technology in the parameters of creation is indisputable. In this vein, digital literature turns readers into experimental creators (Escandell, 2020: 91–103).

All this means that the vision of the digital work can become an apparent obstacle for the reader, who may give up trying to set the digital work in motion. This occurs when the digital base is dissociated from the text by approaching it exclusively with conventional keys. It is therefore a technological dynamism where the literary game also includes a technical game in terms of mastering software and the multiple resources linked thereto. The reader needs to understand digital communicative strategies and the effects they can have on the digital text itself.

Barely a decade ago, Romero-López (2012) proposed a first classification of digital literature insofar as it was handled by the reader. He distinguishes three types of this literature: hypertextual literature, in which the link plays a key role in the creative process; ecphrastic literature, in which text and image form a harmonious whole; and serendipitous literature (serendipity), marked by chance or unpredictability through which the reader can achieve his creative purpose.

In any case, there is no doubt that digital literature, with its markedly creative character, offers the reader many creative possibilities that he will have to experiment with. From the mastery and knowledge of the essence, intention and meaning of the literary message, and from the competence in knowing how to master the techniques of digital creation, a creative experience will emerge, in which reading and digital writing act in parallel and at the same level.

## Poetry in the Context of Digital Literature

The developments in technology have always had a huge impact on every field of human activity. It is therefore necessary to recognise that technology has been and is a key factor to be taken into account in the progress of the history of literature. If the technology of the printing press changed the medium of transmission of literature from oral to written, the Internet allows the poet to approach the reader through digital poetry, whereby the latter can get to know the poem’s setting of movements and sounds beyond its mere representation in a digital environment.

The Internet has provided a new scenario for poetry in two ways: as a means of dissemination and promotion of the poetic text, as well as a creative tool for the design of the literary message. As Torres (2013) points out in this regard, the poetic genre has found in technology ‘a new transtemporal and trans-spatial dimension, expanding its frontiers with surprising and open forms’.

It should be noted that one of the literary genres with the least presence in formal literacy settings has been chosen for this change of formal discourse. The reason for this—or one of them at least—is that the potential reader of digital poetry is an active and immediate recipient, a true performer of the literary work in digital environments. It should be made clear that not everything published on the Internet is digital poetry, since digitisation implies, as we have already said, interaction with the text.

### Towards a Definition of Digital Poetry or Cyberpoetry: Identifying Characteristics and Classification Proposal

Digital poetry is the latest challenge of digital literature in the so-called paraliterary era. In this sense, poetry spontaneously shifts to digitalisation, visuality, fragmentation and, at times, absurdity. As we will see in our study, all this has led to the emergence of poetic practices enabled by technology and for digital media, since today printed texts lose their importance due to the technological development of digital media (Soccavo, 2015). However, there is no doubt that this new poetry, of a markedly electronic nature, whose natural space is the web and which makes the most of the capabilities that the digital medium offers (Saum-Pascual, 2019), must maintain the aesthetics and creativity that are typical of this genre. The reason for this lies in the main characteristic of poetic texts: recipients thereof continue to attach greater importance to the aesthetic value of the linguistic message and to the genre’s ability to move the listener’s mood and its special versatility in renewing language. However, it must be clear that cyberpoetry is not writing a poem and posting it, for example on Twitter.

As a mirror of the most recent human activities and philosophies of society, poetry moves in step with modern technology, which believes in the globalisation of art and literature. Therefore, digital poetry not only breaks all the traditions and limitations of the written text, but also frees itself from the constraints of a certain culture or a certain language. In other words, digital poetry can be described as an electronic, multicultural and multilingual global movement, which can merge the textual and visual elements of poetry with technology (Kovalik and Curwood, 2019: 185–195). It will also be necessary to mention that the ability of digital poetry to consolidate itself as a human activity born within digital culture is its flexibility and lack of concrete form, its willingness to show a strong symbiotic relationship with the technology that hosts it and its ability to adapt to a wide variety of environments for its transmission.

Digital poetry is heir to the aesthetics of the historical *avant-gardes* and, to a greater extent, to the *avant-gardes* of the second half of the 20th century, such as Lettrism, concrete poetry and conceptual poetry (Saum-Pascual, 2019). Lettrism, founded by Isodore Isou in Paris, understood that poetry should be based on the constituent elements of language such as letters and sounds, beyond meaning or semantics, used in a new way that took into account their materiality and not their representational capacity. In the context of lyricism, the notion of *détournement* also arose, in which social messages were appropriated, altered, intervened, subverted or ‘hacked’ (Bentivegna, 2019: 135–156).

Digital poetry has received plenty of names, but one of them that seems very appropriate to the specialised bibliography is cyberpoetry (Escandell, 2019), that is, a form of cyberliterature in which the aesthetic function of language dominates the literary message. Technically, cyberpoetry is characterised by the use of various electronic resources, including the well-known hypertext, two- and three-dimensional animation and more advanced realities such as virtual and multimodal. However, it cannot be forgotten that cyberpoetry, in addition to being associated with the emerging technology of the moment, is above all associated with the biological component of literature, that is, it is created to be enjoyed aloud insofar as it constitutes a full aesthetic experience (Escandell, 2019).

There are three basic characteristics of cyberpoetry: firstly, experimentation, since it seeks the limits of the art of literature and its ultimate aim is to break the rules of this art (Morales-Sánchez, 2019); secondly, collectivisation in digital environments that can be accessed by an unlimited public; and finally, co-creation, given that it is not an individual work but can involve artists from different backgrounds and tendencies who converge in the same text: from the writer himself to the author of the visual animation that surrounds the work (Campos-F.-Fígares, 2021: 7–10; Falguera-Garcia and Selfa-Sastre, 2021: 4–5).

There is a possible classification of the cyber-possibility if we take into account the technology used for its construction. Such classification would be as follows:

Hypertextual poetry is poetry that is linked to another location in the same document or to different documents on the Internet. Although there are fewer examples, it is also the one that uses hypertext for the creation of a poetic work (Morales-Sánchez, 2019).
Animated or moving poetry, also called kinetic poetry (Campos-F.-Fígares, 2021: 9): poetic works where the text’s words are gradually moved or modified, either by the user’s interaction with the text or automatically.
Holopoetry or holographic poetry: This is the name given to texts with poetic content developed through the use of the holographic technique. Thus, the poem is organised in a non-linear way in a three-dimensional immaterial space, changing and conveying different meanings even as the reader looks at it (Morales-Sánchez, 2019).

## Objectives

In order to analyse digital literary reading practices and, specifically, the creative editing of cyberpoems in initial teacher training, our study has two main objectives, listed and explained below.

The first objective of our study concerns the digital literary reading practices to be carried out by student teachers in their initial training. Specifically, this objective would read as follows:

- To select literary texts, specifically poems, to which a digitalisation process will be applied and which, after this process, can be handled through digital tools without losing the original meaning of the poem.

In this vein, the content and nature of the poem upon digitalisation cannot lose its original meaning. Therefore, there is a first phase of selection of texts, which have generally been published on paper.

The selection of poems should take two criteria into account: (a) the authors of the texts selected should be recognised by literary tradition; (b) that they can be worked on in Catalan schools throughout the First Cycle of Primary Education, that is, with students between 6 and 8 years of age.

The second objective concerns the analysis of the digitised poem. In particular, the following objective is pursued as:

- To relate digital literary reading practices to one of the types of cyberpoetry described in the previous section and to analyse them in relation to the categories and analysis processes related to the most important characteristics of digital poetry. These characteristics have to do with the multimodality, hypertextuality and interactivity that are characteristic of digital poetry.

## Research Design and Method

Research design takes into account the data collection techniques, method and analysis categories and processes chosen by a researcher to combine them in a reasonably logical way so that the research problem is handled efficiently.

### Research Design, Participants, and Data Collection Techniques

The aforementioned digital literary reading practices were designed within the subject Didactics of Language and Literature II, 3rd year of the Double Degree in Early Childhood Education and Primary Education at the Faculty of Education of Universitat de Lleida (UdL). In this subject, we work on content and skills related to children’s poetry, its authors in Catalan and the characteristics of digital poetry to be worked on in the classroom in order to develop the reading and literary skills of students aged between 6 and 8.

The research design was carried out as follows. The participants are 30 students who work in groups of 5 people and have received theoretical and practical training in the classroom on what digital literature is, its most important characteristics, the definition of what digital literary reading practices are with examples of this and the types of cyberpoetry that the specialised bibliography contemplates.

They are digital natives, which means that they are familiar with digital tools for the creation of virtual literary reading environments. From this point of view, the participants in this research designed their digital literary reading practices based on the *Genially* application,^1^ an online tool suitable for creating creative, interactive and animated content. This tool allows work based on three basic principles that can be used to create digital literary reading practices. These are the following:

- Animation. *Genially* allows to animate images and graphics through motion. Thus, the creator of the digital content can easily configure entry or exit animations, to indicate just one example. In this sense, it enriches the content created with visual effects that convey the idea of movement. In the specific case of digital literary reading practices, with this application, we create moving images associated with poetic text.
- Interactivity. *Genially* allows also, without the need to have specific programming knowledge, to interact with the poem that it is going to be digitized. The creator of the digital proposal decides where to intervene to create more visual content, which has often movement and, therefore, transmits less saturated information.
- Integration. *Genially* facilitates the integration of information that it is on the Internet or that the user has saved on different platforms. Thus, it can place content from different sources within *Genially* that will continue to work through hyperlinks. We are referring to platforms such as YouTube, Videos, Documents, Gadgets and 3D Images.

Once the proposals have been created in this *Genially* application, the application generates a URL as an electronic address, which we have used to collect the data. The collection of these electronic addresses helps that researcher to access each proposal created for subsequent analysis.

### Method and Categories and Analysis Processes

The research method is defined as the set of techniques that, consistent with the orientation of an investigation and the use of certain tools, will allow obtaining a particular product or result. In our case, we opted for qualitative research, which tends to seek the causes of phenomena in the depth of the interpretations that the subjects make of them. In this way, we have worked with a sample of materials, which has constituted our sample. The qualitative methodology allows us to obtain data from the meanings that each part of the sample wants to convey. This obtaining allows us to establish categories of analysis for each digital practice of literary reading.

The analysis categories to study the digital literary reading practices in question, which is the result of a previous process of digitisation applied to a literary text that was conceived to be read as printed, are the following:

- Multimodality: the resulting digital text presents audio, voice and image modes that refer to the main content(s) of this literary text. These poems contain sensory words easily associated with sounds or images. The potential for digitisation of poems is greater where verses include words that can be quickly and almost involuntarily related to specific images, colours or sounds. These concepts are called sensory words, as they trigger memories related to the senses and evoke and express emotions.
- Hypertextuality: the digital text establishes a relationship that a text b (hypertext) maintains with a previous text a (hypotext) in which it is inserted without being a commentary. One of the characteristics of digital literature is the possibility of including hyperlinks to connect the different intertexts that the poem may include. This way a universe of connection between readings is created.
- Interactivity: the digital text is focused as a game with the explicit participation of the reader. Therefore, it allows for the narrative creation of the reader, as the reader is positioned as a collaborator in the storey, either as an avatar or as an outsider, with a narrative responsibility. They are poems that convey a sense of dynamism/action. In reference to this criterion, we find that certain poems quickly evoke a sense of movement, either because of the rhythm of the poem or because of what the content of the poem expresses. This feature is accentuated when the text contains action verbs.

Therefore, our study, as we have said before, is both qualitative and descriptive, as it aims at showing very specific digital literary reading practices whose main purpose is the creation of virtual environments for working with poetry. It should be noted that the qualitative study is one of the most operational in educational research as it allows for a direct relationship between the researcher and the object of study.

## Results and Discussion

In order for the results and their analysis to be presented, we will consider the two objectives of our research and the three analysis categories and process defined and explained in the ‘method’ section above. As we have already said, this is a qualitative, descriptive study which, it must be said, selects those digital practices that allow us to draw conclusions, as we will discuss later.

### Text Selection

Text selection is related to the level of teaching and learning they are aimed at. In the case of poetry, a genre generally considered eclectic in the school sphere with respect to other genres more commonly worked on in the classroom, such as the narrative genre, it has to present a set of characteristics for students from 6 to 8 years. These characteristics are the following: it allows students to approach their immediate environment through simple verses in their first years of compulsory schooling. The practice of this literary genre should awaken a special motivation among our schoolchildren to learn it, as long as this activity is approached in a playful and attractive way. Secondly, this taste for verses will give rise to poetic texts that will allow the education of the aesthetic sensibility and taste of students who will begin to be motivated by the creation of their first poems based on the correct interpretation of those read in the classroom. This creation can take the form of oral and written productions. Finally, the selected poetry should serve to express one’s own inner world in words that constitute a privileged instrument for the oral and written expression of feelings, emotions and univocal experiences (Selfa and Azevedo, 2013: 58).

The selected poems, the primary basis for applying a digitisation proposal, have very appropriate characteristics that make them especially suitable to be worked on with children between the ages of 6 and 8. From a formal point of view, they are short poems, of no more than two stanzas and with lines of verse generally of less than 8 syllables. They are therefore very suitable for memorisation and subsequent recitation aloud. From a thematic point of view, they deal with realities very close to children of the ages outlined above. As we will see later when we analyse the digitisation proposals applied to these, they refer to realities such as letters, the alphabet, food (tomatoes and bread, for example), animals (frogs, for example) and elements of nature that form part of children’s imaginary (the sun, rain and spring, for example). These are just a few examples, which can be accessed through the hyperlinks below.

### Types of Digital Reading Practices

Generally speaking, in the poems that form the basis of our analysis, kinetic poetry predominates, that is, poetry that produces some kind of movement from the presentation of the digital text to the reader and, above all, in the interaction that the latter has with the poetic text. The poem *Sopa de Lletres* (*Alphabet Soup*) would be a first example of this.^2^ The way in which it is initially presented to the reader is in movement. The letters of the alphabet, which are the main motif of the poem, are presented in twists and turns until they adopt a static position. The same applies to the set of letters below the poem with which the reader can interact to create new words.

A similar case would be that of the digital poem *Cançoneta de les granotes* (Frog song),^3^ in which the reader perceives the movement of the numbers 1, 2 and 3 linked to the numerical concept they present.

The kinetics of the digital text can also be represented by the movement offered by some of the images that refer to the content of the literary text. In the digital text dedicated to letter *F*^4^ and entitled *F*, the pendular movement of the capital letter, adorned with flower attributes, is reminiscent of one of the words that are central to the understanding of the literary message: flower. This movement is the one produced when the flower is shaken by the wind.

This type of kinetic poetry sometimes presents a more static movement, involving a visual transit through the poem and then interacting with it. The poem entitled *Pa amb tomàquet* (Bread and tomato)^5^—a typical breakfast in Catalonia—in the first picture thereof, the reader is informed that in order to play with it, he must pick the picture of one of the three cooks on the title page of the poetic text. Subsequently, a second picture appears in which the reader must fill in missing words in each of the lines of the poem with images that appear visible or hidden in some parts of the kitchen.

There are also some cases of hypertextual poetry, in which the digital poem itself can be used to access other documents posted on the Internet that are related to the content of the poem. This is the case of *Cançoneta de les granotes* (Frog song).^6^ In this one, through the selection of a few words, the reader can travel through the Internet through hyperlinks created to broaden and deepen the meaning of the poem.

The same can be said for the hypertext-based digital text *Aficions,*^7^ in whose verses highlighted words appear and which refer to sound and visual archives that broaden the meaning of the concepts expressed.

We have not found example of holopoem in the group of cyperpoems that form the basis of our study. This may be because the poetry is three-dimensional, very digitally creative and involves a spatial vision that is more suited to adult readers. It should be remembered that, as we have already pointed out, the cyberpoems created here are aimed at students aged between 6 and 8.

### Multimodality

Multimodality aims to facilitate the reader’s digital interaction with the poem. Normally, from a very early age, students are immersed in the digital world, which allows them new ways of reading that modify traditional ways of learning. In the digital proposal *Sopa de Lletres* (see footnote 2), multimodality is provided by the link between the digital text and a sound file located at the bottom right of the text. To access this file, the reader selects the voice icon which takes him/her to a sound video, in which you can discover all the letters of the alphabet and see them appear in movement in full colour.

Similarly, in initial interface of *Pa amb tomàquet* (see footnote 5), a musical icon appears next to the title of the poetic text, which again allows us to highlight the multimodality of the poetic text. In this case, by selecting the aforementioned icon, the reader accesses a Youtube video in which the reader mediator addresses the reader of the poem, explaining how this breakfast is prepared through gestures and the use of the narrative voice.

Multimodality is also expressed by moving and sometimes static images representing key words in the digital text. In *F* (see footnote 4), the word *ferida* (wound) is healed by a band-aid and, in the last verse of the text, the word *petó* (kiss) is represented by red lips that prefigure the image of a kiss in movement.

### Hypertextuality

The digital text is the hypotext, i.e., the starting point that refers to new texts, called hypertexts, which provide information of all kinds related to the main text. Hypertextuality could be said to be a static function, as one simply shifts from one digital stage to another digital stage. As mentioned above, there is an informational connection between the two. This can be found in *Pa amb tomàquet*,^8^ by the universal Catalan poet Miquel Martí i Pol. In the initial presentation of the poem, next to the author’s name, there is an icon that refers to a call for information. When the reader selects it, they are taken to an Internet page where they can zoom in and find out in depth who the author of the poem is and what kind of poetry he has written. In this case, it is thus a matter of extending information through a digital narrative text.

In other cases, hypertextuality, as outlined above, allows the reader to virtually scroll through other links on the Internet to learn more about the words in the digital text. In *Cançoneta de les granotes*,^9^ the digitisation proposal allows, in the third verse, access to an image that presents the word *estiu* (summer) and, in other cases, access to the visualisation of writing in capital letters, typical of the early stages of learning to read and write, of the word *sol* (sun).

### Interactivity

Poetry is a game. As such, it allows an interaction that places the reader as a creator of new poems, enabling new learning in accordance with his age and reading and literary competence. In *Sopa de Lletres* (see footnote 2) above, we can see how the reader can create new words without losing the original meaning with which the poem was created. To do this, the digital editor of the poem warns the reader that he can move the letters and lines and create new words, as well as compose his own poem. Letters and verses that are not needed can be left out of the yellow box. This is the poetic and digital game that is proposed. We see how the initial word *mitjó* (sock) which appears in the initial proposal of the author of the poem, when the reader plays with it, he can modify it and obtain a new word, as in this case is *águila* (eagle).

At other times, it will not be a matter of creating new words, but inviting the reader to create a new poem by moving the words of the poem. As can be seen in the poem entitled *F* (see footnote 4), the words written in capital letters can be moved and combined to obtain a new poem and thus give the text a new poetic meaning without losing the spirit of the text.

Another example of the creation of a new poem from the initial stage offered by the digital text is also the poem *F*,^10^ to which a different digitisation proposal than the one presented above is applied. In this case, the reader can compose new verses, different from the original version created by the author of the poem, by moving the words and placing them where they can make sense.

As mentioned above, interactivity requires manipulating the digital text. This manipulation can also be associated with images that help to better understand the content of the manipulation, as we can see, for example, in *Pa amb tomàquet* (see footnote 11). The reader of the text, once he has accessed the stage with which he can play with the text, will be able to complete the missing words in the poem by moving images that refer to these words. These images are visible at first glance, as in the case of the ham on the table, or they may be hidden in the kitchen cupboards or fridge. The reader of the text will have to digitally open the doors of these cupboards and fridges to find the images that will later be placed in the digital poem.

The evaluation of the digital practices of literary reading was carried out with an evaluation rubric, which is a tool that helps to evaluate the students’ learning, making the students themselves also know their successes and errors through self-assessment. This rubric was given to the students before starting the creation project and has two elements: a vertical column, which contemplates the evaluation criteria of said creation and which, in our case, have to do with the categories and analysis processes; a horizontal column with the quality grades of those criteria. The rubric used was the following (Table 1).

**Table 1:** Evaluation rubric.

Indicators	Very good	Good	Regular	Insufficient
Analysis categories and processes				
Multimodality				
Hypertextuality				
Interaction				

Source. Own creation.

The results that we have obtained and that we have discussed in the theoretical framework of this research allow us to affirm that, in general terms, there are studies related to cyberpoetry, as a branch of digital literature, that describe and analyse the digital environments in which cyberpoems are published. They are studies that research the work of young authors and that the literature calls *Millennials*. They are called as ‘childrens of Instagram’ (Sánchez García and Aparicio Durán, 2020: 41–53). Many of them have a place in the digital channels of literary communication that are widely distributed on the Internet. An example of what was said is *Wattpad* (Falguera-Garcia and Selfa-Sastre, 2021). Some have even been recognised with national and international literary awards. However, the implication of our study goes beyond analysing creators of digital literature. In our case, we are talking about literary reading mediators who create from digital tools, as is the case with *Genially*. Starting from a text by another author, the teacher in initial training applies a digitization process to the literary text in order to be able to play, interact and experiment with it. Only in this way is the interaction effective, practical and the transition between the paper text and the digital text are completely assured without altering its message.

## Conclusion

This article aims at exploring the role of digital literature in initial teacher education. From an eminently theoretical perspective, we identify the concept of digital literature in current literacy contexts and, specifically, what we understand by digital poetry from the point of view of creativity. The final objective of this work is precisely this to show models of digital literary reading practices that can be carried out in the classroom, starting from the premise that these are texts selected for students aged 6 to 8 years old and that they are still in the initial stages in the formation of their literary competence.

From our study we conclude that, in the first place, the selection of texts published in printed anthologies follows the criteria of children’s poetry: poems with short verses and few stanzas, close to the oral language and therefore easy to memorise and later to declaim. These poems thematically refer to realities that are well known to students aged 6 to 8: nature, animals, food and childhood experiences. From this point of view, the digitisation proposal applied to these poems will take these elements into account and, therefore, digital play with this poetry will be the common denominator of the digital practices analysed in our study.

On the other hand, if we look at the classification of the digital practices presented, which are the result of a previous digitisation of poetry that has been initially conceived to be declaimed, the vast majority of these are kinetic poems or poetry in movement. These are digital proposals that allow experimentation through the movement of letters, words and images that result in new poems with a different meaning to the original poem, although the initial essence of the poem is not lost, i.e., its structure is practically the same.

Secondly, in terms of the categories of analysis of digital poetry practices, interactivity, understood as a game, as experimentation and as a new way of learning concepts, stands out in the first place. Through digital tools, the digital reader can move, change and transform the reality of the poem they are working with.

Multimodality is also present in the digital proposals under analysis. In these cases, when we speak of multimodality, we are referring to sound files that reproduce sounds related to the key words of the poem. These are characteristic and well-defined sounds that facilitate the understanding of the poem and are usually hosted on well-known platforms such as Youtube. Similarly, multimodality is also related to moving images, which again, link to key words in the poetic text.

Finally, there are also digital practices of literary reading of poems in which hypertextuality prevails in the digitisation proposal. These are less elaborate, more primitive practices that basically establish a relationship between a hypertext and a previous text, the initial poem, which we call hypotext. In the examples shown in this text, hypertextuality is often used to expand info on the author of the initial poem. Its function is basically informational.

Finally, our work has its limitations, which are inherent to the selection of a very specific sample of texts. Among these limitations, we highlight the following: (a) one of them has to do with the selection of texts in a particular language: Catalan. We have already said that student teachers in initial teacher training prepare these digital practices for a specific school context, schools in Catalonia. While this must be the case because it is a reality that determines the choice of literature to work with, future studies can compare the creation of digital practices in Catalan with other languages. Secondly (b), the age groups targeted by digital practices also condition the preparation of these practices. These are early ages, where literary and digital competence are being constructed. These are therefore sometimes flat proposals which, with a greater degree of elaboration, can be applied at older ages. Finally (c), the digital practices analysed have an impact on reading comprehension of the text and very little on the written elaboration of new messages. From this point of view, some kind of interaction with the text could have been thought of, involving the rewriting of the text not only by moving images or letters, but also by creating more elaborate messages.

All of this do not detract from the digitalisation proposals presented, understood within the context of the digital literacy that takes place in schools that cater for children of an early age, as are the targets of the digital practices we have analysed. Digital literature is emerging strongly from creative, original perspectives that facilitate access to the text. This way, digital literature and, specifically, digital literary reading practices, as particular manifestations of the former, are conceived a way of presenting and working with the initial reader in a literary genre very close to oral language, which offers many possibilities for interaction, play and learning. The objective of the digital texts under analysis in this study is to learn from poetry, through playful environments previously created from the premise of digital creativity.

## Data Availability Statement

The original contributions presented in the study are included in the article/supplementary material, further inquiries can be directed to the corresponding author.

## Author Contributions

MS is responsible for the theoretical framework and the design of the research that we present in this research. EF is responsible for the practical implementation of digital literary reading practices in initial teacher training. All authors contributed to the article and approved the submitted version.

## Funding

This research has been funded by the AGAUR (Agència de Gestió d’Ajuts Universitaris i de Recerca) under Grant 2020 ARMIF00020.

## Conflict of Interest

The authors declare that the research was conducted in the absence of any commercial or financial relationships that could be construed as a potential conflict of interest.

## Publisher’s Note

All claims expressed in this article are solely those of the authors and do not necessarily represent those of their affiliated organizations, or those of the publisher, the editors and the reviewers. Any product that may be evaluated in this article, or claim that may be made by its manufacturer, is not guaranteed or endorsed by the publisher.

## Footnotes

^1^ https://genial.ly/es/

^2^ https://view.genial.ly/61bf5b75e6cbe90e3841ff7c/interactive-image-interactive-image

^3^ https://view.genial.ly/61ba10d1295ff00de456c1e2/interactive-image-canconeta-de-les-granotes

^4^ https://view.genial.ly/61c345f84d9c8c0df24d07e0/interactive-content-f

^5^ https://view.genial.ly/61c2fee64d9c8c0df24cfc8f/interactive-content-poemadigitalcostafornonsgallegosole

^6^ https://view.genial.ly/61c3057a4d9c8c0df24cfddd/interactive-image-canconetadelesgranotes

^7^ https://view.genial.ly/61bc52ad5ea1040d618602c6/interactive-content-poema-digital

^8^ https://view.genial.ly/61c2fee64d9c8c0df24cfc8f/interactive-content-poemadigitalcostafornonsgallegosole

^9^ https://view.genial.ly/61c3057a4d9c8c0df24cfddd/interactive-image-canconetadelesgranotes

^10^ https://view.genial.ly/61c20f6461aa590e006ef6a0/interactive-image-f-salvador-comelles
